# Analysis of liver injury factors in 332 patients with COVID-19 in Shanghai, China

**DOI:** 10.18632/aging.103860

**Published:** 2020-10-01

**Authors:** Hongying Guo, Zhengguo Zhang, Yuyi Zhang, Yu Liu, Jiefei Wang, Zhiping Qian, Ying Zou, Hongzhou Lu

**Affiliations:** 1 Department of Severe Hepatopathy, Shanghai Public Health Clinical Center, Fudan University, Shanghai, China; 2 Department of Infectious Disease and Immunology, Shanghai Public Health Clinical Center, Fudan University, Shanghai, China

**Keywords:** coronavirus disease 2019, COVID-19, SARS-CoV-2, liver injury, medication

## Abstract

Objective: We analyzed clinical parameters and risk factors for coronavirus disease 2019 (COVID-19)-related liver damage.

Results: Of the 332 COVID-19 patients, 306 and 26 were included in the non-critical and critical groups, respectively. The median time from onset to admission was 4.0 (2.0–8.0) days. Of the 332 COVID-19 patients, 98 (29.5%) were admitted with liver injury. The rates of aspartate transaminase, γ-glutamyl transpeptidase, and total bilirubin abnormalities were higher in the critical group than in the non-critical group. The patient’s sex, COVID-19 severity, and a low liver CT density strongly correlated with liver injury (ORs: 2.936, 6.543, and 3.387, respectively). Statistical analysis on drug administration after admission showed that the usage rates of lopinavir/ritonavir, glucocorticoids, and thymopeptides were significantly higher in the abnormal group than the normal groups (*p*<0.05).

Conclusions: Though not severe, the liver injury among COVID-19 patients was pervasive. Being male, COVID-19 severity, low CT density, and medications may be risk factors for liver damage. Following recovery, liver function gradually returns to normal.

Methods: This retrospective study screened 332 confirmed COVID-19 patients from January 20 to March 13, 2020. Liver indicators were evaluated on admission. The risk factors, medications, and the dynamic change of liver functions were analyzed.

## INTRODUCTION

Coronavirus disease 2019 is caused by a novel beta coronavirus, which has now been named severe acute respiratory syndrome coronavirus 2 (SARS-CoV-2) and has led to the infection of millions of people [1]. The number of COVID-19 patients is still rapidly increasing worldwide. Although most COVID-19 patients have a mild disease (81%), some will develop a severe illness and require oxygen therapy (14%) and approximately 5% will require intensive care unit treatment [2, 3]. The mortality of COVID-19 is about 3.7% [4], which has resulted in thousands of deaths globally.

The main manifestations of COVID-19 include fever, fatigue, and dry cough. Nasal congestion, runny nose, sore throat, myalgia, and diarrhea have also been reported in a few cases. Severe cases mostly developed dyspnea and/or hypoxemia after one week. In severe cases, patients can progress rapidly to acute respiratory distress syndrome, septic shock, metabolic acidosis that is difficult to correct, coagulopathy, and multiple organ failure [5]. Recent studies on COVID-19 have shown that the incidence of liver injury ranged from 14.8–53%, and was mainly indicated by abnormal alanine aminotransferase (ALT) and aspartate transaminase (AST) levels, accompanied by slightly elevated total bilirubin (TB) levels [3, 5–7]. Albumin levels are decreased in severe cases, with levels around 26.3–30.9 g/L [8]. Some data have shown that serum γ-glutamyl transpeptidase (γ-GT) levels increase in severe cases and serum ALT levels are normal in both mild and severe cases [9].

It has been shown that, like SARS-CoV, SARS-CoV-2 also uses ACE2 as its entry receptor [10]. Chai et al. found that both liver cells and bile duct cells express ACE2 [11], which might be the main reason that some COVID-19 patients suffer from liver injury. In fact, one study reported that the liver injury observed in COVID-19 patients might be caused by lopinavir/litonavir, which is used as an antiviral for the treatment of SARS-CoV-2 infection [12]. However, the study is limited by the small number of patients taking lopinavir/litonavir and with only 8 patients in the normal liver function group. Thus, further studies on a larger population are necessary to confirm this observation.

In our study, we analyzed liver function parameters and risk factors in 332 patients with COVID-19 on admission in Shanghai, China. Furthermore, we screened 234 patients who had normal liver function on admission but later 92 patients developed liver injury. We evaluated the influence of medications on the liver, including traditional Chinese medicines. Through the analysis of clinical data, we identified factors of liver injury in patients with COVID-19.

## RESULTS

### Baseline parameters of patients with COVID-19 when admitted

There were 332 patients with COVID-19 included in this study, of whom 174 (52.4%) were male and 158 (47.6%) were female, with a maximum age of 88 years, a minimum age of 15 years, and a median age of 50 (36–64) years. Based on disease severity, patients were divided into a non-critical group (306 cases including mild and moderate type) and a critical group (26 cases including severe and critical type). The median time from onset to admission was 4.0 (2.0–8.0) days.

Of the 332 patients, 216 (65.1%) patients showed decreased pre-albumin (<180 mg/L), mean: 115.5±37.3 (mg/L); 143 patients (43.1%) showed decreased albumin (<40 g/L), mean: 36.7±2.9 (g/L). Decreased pre-albumin was common in COVID-19 patients. A total of 127 (38.3%) patients showed various levels of lactate dehydrogenase (LDH) elevation (>250 U/L), with a median of 314 (276–385) U/L. LDH elevation was more common in the critical group, and the magnitude of the elevation was greater. Only 1 of the 26 critically ill patients had a normal LDH value at admission; the median value in the critical group was 406 (357–508) U/L.

**Figure 1:**
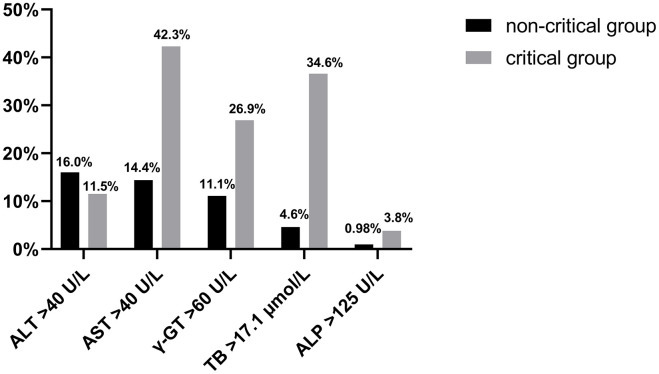
**The rate of ALT, AST, γ-GT, ALP, and TB abnormalities is shown in the critical and non-critical group.**

Ninety-eight (29.5%) of the 332 COVID-19 patients were admitted with liver injury based on baseline liver function tests, including 18 (69.2%) in the critical group and 80 (26.1%) in the non-critical group. There was a significantly higher probability of liver injury in the critical group (*p*<0.0001). The rates of AST, γ-GT, and TB abnormalities were significantly higher in the critical group than in the non-critical group (Figure 1). The degree of concurrent liver injury was less severe in the COVID-19 patients; the mean of ALT, AST, ALP, γ-GT, and TB did not exceed the upper limit of normal value (ULN) by twofold (Table 1).

**Table 1:** Abnormality among main indicators of liver injury on admission.

	**ALT (U/L)**	**AST (U/L)**	**ALP (U/L)**	**γ-GT (U/L)**	**TB (μmol/L)**
Abnormal rate (%)	52, 15.7%	55, 16.6%	4, 1.2%	41, 12.3%	23, 6.9%
Mean ± SD	64.5±22.4	64.0±34.6	140.5±14.2	116.1±57.7	23.4±6.7
Max	138	266	159	402	47.1

Compared to the group with normal liver function, the proportion was large for the male patients with liver injury (73.5% vs. 43.6%, *p*<0.0001), and difference for those with chronic underlying diseases (34.7% vs. 33.8%, *p*=0.02). The occurrence of low liver CT density was more common in the abnormal group (34.7% vs. 13.2%, *p*<0.0001). As expected, the chronic alcohol drinkers tend to have higher liver injury (8.2% vs. 1.7%, *p*=0.004), the same as the patients with critical illnesses (18.4% vs. 3.4%, *p*<0.0001). The study also showed that the patients with previous combined chronic liver disease had larger proportion in the abnormal liver function group than those in the normal group, but there were no significant differences. In contrast, there were no significant differences in age, CT suggestive of gallbladder stones or cholecystitis, time from disease onset to admission, or out-of-hospital antimicrobial drug use between the two groups, as shown in Table 2.

**Table 2:** Risk factors for liver injury in the normal and abnormal liver function groups.

	**abnormal (n=98)**	**normal (n=234)**	**statistics**	***p-*value**
Sex (%)	72, 73.5%	102, 43.6%	X^2^=24.724	<0.0001
Age	54 (39–64.3)	48 (34.8–63.3)	Z=-1.66	0.097
Complicating diseases other than chronic liver disease (%)	34, 34.7%	79, 33.8%	/	0.027
Chronic liver disease (%) *	7, 7.1%	9, 3.8%	/	1.632
Low liver CT density (%)	34, 34.7%	31, 13.2%	X^2^=20.177	<0.0001
Gallbladder stones or cholecystitis on CT (%)	5, 5.1%	9, 3.8%	/	0.564
Drinking history >5 years (%)	8, 8.2%	4, 1.7%	X^2^=8.233	0.004
Time from onset to hospital admission (day)	5 (3–7)	4 (2–8)	Z=-1.061	0.288
Out-of-hospital antimicrobial drug use (%)**	27, 27.6%	47, 20.1%	X^2^=2.223	0.136
COVID-19 severity (%)	18, 18.4%	8, 3.4%	X^2^=21.384	<0.0001

*Chronic liver diseases involved in this study include autoimmune hepatitis, positive hepatitis B markers, and positive hepatitis C antibodies.

**The top three used antimicrobial drugs in the hepatic injury group were moxifloxacin alone in 9 cases, cephalosporins alone in 5 cases, and cephalosporins combined with respiratory quinolones in 3 cases. The top three used antimicrobial drugs in the normal liver function group were moxifloxacin alone in 13 cases, levofloxacin alone in 11 cases, and cephalosporins in 6 cases.

**Figure 2:**
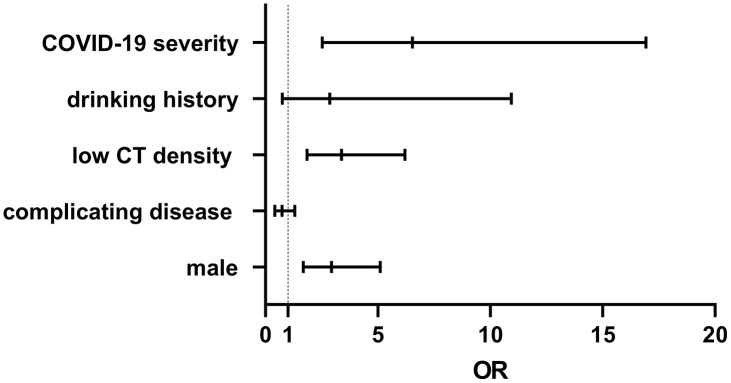
**A patient’s sex, COVID-19 severity, and low liver CT density, strongly correlate with liver injury, with OR values of 2.936, 6.543, and 3.387, respectively.**

Many risk factors such as patient’s sex, COVID-19 severity, drinking history, low liver CT density, and complicating diseases were analyzed by binary logistic regression. The results revealed that liver injury strongly correlate with patient’s sex (OR 2.936, 95% CI 1.688–5.106, *p*<0.0001), COVID-19 severity (OR 6.543, 95% CI 2.528–16.93) and low liver CT density (OR 3.387, 95% CI 1.85–6.201, *p*<0.0001, Figure 2).

### Analysis of abnormal liver function during hospitalization

Of the 332 COVID-19 patients, 98 (29.5%) were admitted with liver injury, based on baseline liver function examination, and 234 patients had normal liver function. Of those 234 patients, 92 (27.7%) presented with liver damage after admission. Thus, 190 (57.2%) of the 332 patients had liver function abnormalities. The 234 patients were divided into two groups (normal and abnormal liver function). We analyzed the medications of the patients, including antiviral drugs (lopinavir/ritonavir, arbidol, hydroxychloroquine, and darunavir cobicostat), antibiotics (azithromycin, quinolones, and cephalosporin), herbal (Lianhua Qingwen granules, Shufeng Jiedu capsules, and Xuanfei Zhike mixture), glucocorticoids, thymopeptides, interferon spray, sedatives and hypnotics, analgesic-antipyretic, antilipemic agents, hypotensor, hypoglycemic agents, and drugs for coronary heart disease. All the drugs used after admission were compared between the two groups. The results showed that the usage rates of lopinavir/ritonavir, glucocorticoids, and thymopeptides were higher in the abnormal liver group of patients (*p*<0.05). Further analysis of the two groups revealed that the proportion of male patients in the abnormal liver function group was higher (Table 3).

**Table 3:** Medication in abnormal and normal groups during hospitalization.

**Parameter**	**Abnormal (n=92)**	**Normal (n=142)**	***p-*value**
Age (Years)	51(15–85)	46(16–81)	0.1579
Sex (male %)	55, 59.8%	47, 33.1%	0.0011
ALT (U/L)	49.0 (7.0–310.0)	15.0 (5.0–39.0)	<0.0001
AST (U/L)	31.0 (10.0–488.0)	18.0 (9.0–36.0)	<0.0001
ALP (U/L)	59.5 (23.0–430.0)	53.0 (13.0–103.0)	<0.0001
γ-GT (U/L)	38.0 (8.0–235.0)	17.0 (9.0–58.0)	<0.0001
TB (**μ**mol/L)	10.1 (2.3–91.1)	8.0 (3.3–16.8)	<0.0001
Lopinavir/ritonavir (%)	48, 52.2%	29, 20.4%	<0.0001
Arbidol (%)	42, 45.7%	60, 42.3%	0.6858
Hydroxychloroquine (%)	32, 34.8%	48, 33.8%	0.8886
Darunavir cobicostat (%)	5, 5.4%	16, 11.3%	0.1620
Azithromycin (%)	2, 2.2%	9, 6.3%	0.2084
Quinolones (%)	36, 39.1%	41, 28.9%	0.1179
Cephalosporin (%)	9, 9.8%	10, 7.0%	0.4711
Glucocorticoids (%)	24, 26.1%	10, 7.0%	<0.0001
Thymopeptides (%)	21, 44.6%	37, 26.1%	0.0044
Interferon spray (%)	76, 82.6%	110, 77.5%	0.4083
Sedatives and Hypnotics (%)	25, 27.2%	31, 21.8%	0.3521
Analgesic-antipyretic (%)	23, 25%	21, 14.8%	0.0601
Antilipemic agents (%)	7, 7.6%	6, 4.2%	0.3813
Hypotensor (%)	20, 21.7%	21, 14.8%	0.2176
Hypoglycemic agents (%)	11, 12.0%	11, 7.7%	0.3595
Drug for coronary heart disease (%)	12, 13.0%	15, 10.6%	0.6759
Lianhua Qingwen granules (%)	5, 5,4%	4, 2.8%	0.3209
Shufeng Jiedu capsules (%)	34, 37.0%	37, 26.1%	0.0825
Xuanfei Zhike mixture (%)	13, 14,1%	14, 9.9%	0.4025

Non-normally distributed variables were analyzed using Mann-Whitney U test.

Frequencies and proportions were analyzed using the Fisher's exact test.

### Dynamic changes in liver function in patients with COVID-19 accompanying abnormal liver function

The study included 332 patients with COVID-19 and 98 patients with liver injury at baseline, of which 25 (25.5%) had improved liver function at follow-up and 73 (74.5%) had worsened liver function. There were 92 cases of liver injury during hospitalization, of which 41 cases (44.6%) showed improvement in liver function at follow-up, 51 cases (55.4%) showed further deterioration in liver function and 17 cases (18.5%) showed liver injury before discharge. The median time to develop liver injury was 8 (4–13) days; the median time to reach peak liver injury was 11 (8–16) days. Of the 190 patients who developed liver injury as of March 13, 179 patients recovered and were discharged from the hospital; 149 (83.2%) of them had improved liver injury. The summary tests for abnormal liver function, peak liver function, and pre-discharge liver function are shown in Table 4.

**Table 4:** Dynamic changes in liver function indicators in patients with liver injury.

	**Firstly abnormal liver function indicator (n=190)**	**Peak liver function indicator (n=173)**	Pre-discharge liver function indicator (n=179)*
Course of a disease (day)	8 (4–13)	11 (8–16)	19 (15–25.5)
ALT			
Abnormal number (%)	108, 56.8%	129, 74.6%	80, 44.7%
Median (quartile) (U/L)	55 (47–72.8)	72 (55.5–99.5)	58 (48–70.8)
Max	310	426	224
AST			
Abnormal number (%)	83, 43.7%	86, 49.7%	21, 11.7%
Median (quartile) (U/L)	52 (45–71)	59 (49–82)	49 (42–61.5)
Max	488	266	122
ALP			
Abnormal number (%)	7, 3.7%	8, 4.6%	1, 0.56%
Median (quartile) (U/L)	144 (127–220)	130.5 (127.5–199.5)	/
Max	430	650	138
γ-GT			
Abnormal number (%)	60, 31.6%	77, 44.5%	47, 26.3%
Median (quartile) (U/L)	96 (75–131.3)	105 (78.5–128)	86 (73–104)
Max	402	1135	204
TB			
Abnormal number (%)	58, 30.5%	55, 31.8%	168.9%)
Median (quartile) (μmol/L)	22.2 (19.9–27.0)	26.1 (21.4–31.6)	21.2 (19.7–29.5)
Max	91.1	91.1	45.6

* n=179, excluding 8 cases which had not discharged and 3 deaths as of 13 March 2020.

### Follow-up data on liver function in patients with COVID-19 two weeks after hospital discharge

**Figure 3:**
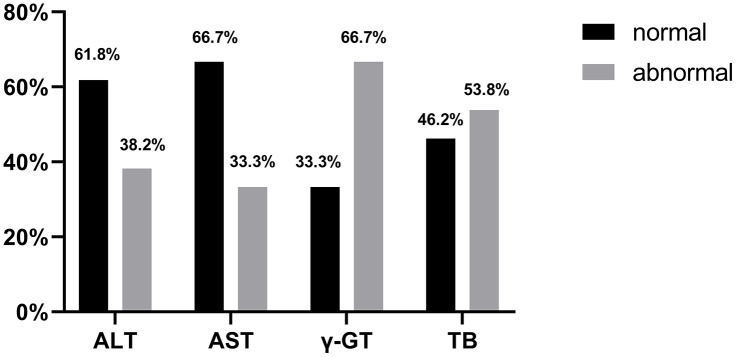
**Proportion of patients with normal ALT, AST, γ-GT and TB at 2 weeks post-discharge follow-up.**

By 13 March 2020, 109 patients with liver dysfunction discharged from hospital were followed up after two weeks. The follow-up data showed that out of the 68 patients with ALT abnormalities, the value of 42 patients returned to normal (42/68, 61.8%); out of the 18 patients with AST abnormalities, 12 patients returned to normal liver function (12/18, 66.7%); out of the 18 patients with γ-GT abnormalities, 6 patients returned to normal (6/18, 33.3%); out of 13 patients with TB abnormalities, 6 patients returned to normal (6/13, 46.2%). There was only one patient with elevated ALP at discharge, whose data were not followed up (Figure 3).

## DISCUSSION

SARS-CoV, MERS-CoV, and SARS-CoV-2 can cause respiratory, intestinal, hepatic, and neuronal diseases and may lead to acute respiratory distress syndrome (ARDS), multiple organ failure (MOF), and even death in severe cases [13–15]. Studies have indicated that patients infected with any of these three viruses may develop liver injury. Research has shown that the level of liver damage is correlated with disease severity, and patients with severe disease have more severe liver damage than patients with mild disease [2, 5, 7, 8, 12]. Among the 332 patients with COVID-19 included in our study, 190 (57.2%) patients presented with liver injury on admission and during hospitalization. The incidence of the liver injury was consistent with a previous study [12]. From the 332 patients with COVID-19, we found that 98 (27.3%) patients presented with liver injury on admission, which is consistent with previous studies [8, 16]. In our study, the critical group included 18 patients (69.2%), while the non-critical group included 80 (26.1%) at admission. This suggests that critical patients were more likely to suffer from liver injury. A previous study showed the presence of an inflammatory cytokine storm in severe COVID-19 cases [17], however, whether this results in liver damage in such patients remains to be investigated. Upon further analysis of the data on risk factors for liver injury, we found that patient’s sex, COVID-19 severity, and low liver CT density were risk factors for liver injury. The higher degree of liver damage in males in this study could be related to a higher proportion of men among critically ill patients. The prevalence of liver damage in critically ill patients supports the view that COVID-19 is a disease that involves multiple organs; and, presumably, the severity of the disease itself plays a major role in liver injury. The previous study indicated that SARS patients with HBV/HCV infection were more prone to develop liver damage and severe hepatitis [18]. In the present study, the history of chronic liver disease was not a risk factor in the patients with COVID-19. The single-factor analysis revealed that the liver injury was related to complicating disease and drinking history, though it is not further confirmed by the results from the multi-factor regression analysis, it might be important for clinicians to be aware of these cases.

Comparing to chronic liver disease, the results of postmortem biopsies in COVID-19 patients showed moderate microvascular steatosis and mild lobular and portal activity in the liver. It was unclear whether the steatosis of liver cells caused by the SARS-CoV-2. Is the liver damage likely caused by SARS-CoV-2 infection or drug-induced hepatotoxicity? Previous studies suggested that antibiotics (macrolides, quinolones), antivirals (ribavirin), steroids, and other drugs used for the treatment of SARS patients may result in liver damage [19, 20]. Many medications including antiviral drug, antibiotics, herbal, glucocorticoids, thymopeptides, interferon spray, sedatives and hypnotics, analgesic-antipyretics, antilipemic agents, hypotensor, hypoglycemic agents, and drugs for coronary heart disease come with warnings of potential liver injury. These drugs are potential causes of liver injury during COVID-19 [3]. In our study, 234 patients who presented with normal liver function on admission gradually developed liver injury. Our results showed that 92 (39.3%) patients suffered from liver impairment during hospitalization, and so the medications were analyzed among these 234 patients. Liver injury was influenced by some medications such as lopinavir/ritonavir, glucocorticoids, and thymopeptides. Lopinavir/ritonavir is currently considered a therapeutic agent that may have some efficacy against COVID-19, and our results suggest that it is associated with liver damage. In addition to liver damage, nausea, vomiting, and diarrhea, other adverse reactions are also common. It is recommended to balance the benefits for the patients of the medicine before clinical application and use cautiously. Close monitoring of liver function is recommended during medication, with a high alert for the occurrence of liver damage. Recently, one study reported that liver injury observed in COVID-19 patients might be caused by lopinavir/litonavir [12], there were just 8 patients in the normal liver function group. To reach a more accurate conclusion in our study we increased the population size and analyzed more drugs. Patients enrolled in this study were treated with a small dose of glucocorticoids (methylprednisolone 20–40 mg/d for ≤5 days) when chest CT indicated rapid imaging progression. Whether the occurrence of liver damage is related to the glucocorticosteroids themselves or to exacerbation of the disease requires further validation in more designed controlled trials.

It has been shown that SARS-CoV-2 also uses ACE2 as its entry receptor [10]. Chai et al. found that both liver cells and bile duct cells express ACE2 [11]. Bile duct epithelial cells are known to play important roles in liver regeneration and immune response. Based on this, it is possible that the liver injury seen in COVID-19 patients may be due to damage to bile duct cells, but not liver cells, caused by the virus infection. Our findings indicated that about 70% patients had high levels of ALT, which suggest liver cell injury. More interestingly, 44.5% and 31.8% patients had high levels of γ-GT and TB respectively, which indicates injury to bile duct cells. Although we could not confirm whether liver damage was caused by the coronavirus infection or drug induced, our findings indicate that liver damage includes both damage to liver cells and bile duct cells. Thus, further research is necessary to explore the mechanism of liver injury in COVID-19 patients. One study showed abundant SARS-CoV-2 viral particles were observed in cytoplasm of hepatocytes in two COVID-19 cases, which could suggest the SARS-CoV-2 infection contributed to liver injury [21]. In the present study, although we found that liver injury was common in COVID-19 patients, liver injury was generally mild, and no cases of liver failure occurred. Moreover, following the disease recovery, liver function gradually normalizes, which suggests that the liver damage in patients with COVID-19 was temporary. It should be noted that 17 (5.1%) of the 332 patients had their first liver injury before discharge, which is a reminder of the need to monitor liver function changes during the follow-up of COVID-19 patients after hospital discharge. We followed up the data of these patients with abnormal liver tests two weeks after discharge, nearly half of them still had not a full recovery, suggesting the clinicians should pay attention to these cases.

Our study has some limitations. Firstly, this was a single-center retrospective study, which was limited by a small sample size. Thus, a future multi-center study is required in order to validate the predicting factors identified in this study. Secondly, further prospective clinical studies should be designed to investigate whether use of specific medications can cause liver damage. Thirdly, the link between liver damage and inflammatory cytokines should be investigated in the future to elucidate the mechanism of liver dysfunction in patients with COVID-19. Additionally, we analyzed whether liver CT density was low or not, but we did not include the detailed CT density values. We did not analyze whether the elevated AST was caused by liver damage, hypoxia, or cardiac impairment in this small proportion of the patients.

The liver injury among COVID-19 patients was common, following recovery, liver function gradually returns to normal, which means the liver damage is temporary. Our study should prompt clinicians to pay attention to monitoring the occurrence of liver injury and the causes of liver injury occurring in different courses of the disease. At the time of admission, for critically ill patients, the clinicians should monitor for multi-organ impairment, including liver function. During hospitalization, physicians should also put focus on the effects of medication on liver function, especially due to drugs associated with liver damage.

This study was supported by Fudan University (IDF162005); Shanghai Public Health Clinical Center (2020YJKY01), and a hospital-level project by Shanghai Public Health Clinical Center (KY-GW-2018-18) and the National Science and Technology Major Project of China (2018ZX10302206).

## MATERIALS AND METHODS

### Study population and design

There were 332 confirmed cases of COVID-19 in Shanghai from Jan 20 to Mar 13, 2020 included in this retrospective study. We analyzed the clinical data and conventional liver parameters of the 332 patients. COVID-19 was diagnosed on the basis of the WHO guidance [22]. A confirmed case of COVID-19 was defined as a positive result on high-throughput sequencing or real-time reverse-transcriptase–polymerase-chain-reaction (RT-PCR) assay of nasal or pharyngeal swab specimens [5]. Abnormal liver function was diagnosed if it met one of these conditions: ALT >40 U/L, AST >40 U/L, Alkaline phosphatase (ALP) >125 U/L, γ-GT >60 U/L, or TBil >17.1 μmol/L. This research project was performed in accordance with the principles of the Helsinki Declaration II and approved by the hospital’s ethics committee.

### Data collection

Clinical demographical records and laboratory tests were reviewed. Clinical indicators included sex, age, clinical performance, and factors of liver injury. Laboratory tests included ALT, AST, ALP, γ-GT, TB, and serum albumin. The use and course of medications were extracted from medication records.

### Statistical analysis

Statistical analysis and plotting were carried out with SPSS 22.0 (IBM, Armonk, NY, USA) and GraphPad prism 8.0 (GraphPad Software, Inc., La Jolla, CA, USA). All variables were recorded and calculated at the first day of hospitalization or when indicated. Continuous variables were analyzed using t-test analysis of variance for continuous normally distributed variables, or the Mann-Whitney U test for non-normally distributed variables, and are reported as means or medians (quartile). Categorical variables were analyzed using the chi-square test or Fisher's exact test, as appropriate, and are expressed as frequencies and proportions. Binary logistic regression analysis was used for modeling the relationship between liver injury events and clinical characteristics. All comparisons were 2-tailed, and p<0.05 was considered statistically significant.

### Ethics statement

All participants provided written informed consent during their admission. The study protocol and informed consent documents were reviewed and approved by the Ethics Committee of Shanghai Public Health Clinical Center, Fudan University.
